# Is total hip arthroplasty safely performed in lung transplant patients? Current experience from a retrospective study of the Zurich lung transplant cohort

**DOI:** 10.1186/s13037-016-0105-x

**Published:** 2016-07-15

**Authors:** Jürgen W. Schmitt, Christian Benden, Claudio Dora, Clément M.L. Werner

**Affiliations:** Department of Trauma Surgery, University Hospital Zurich, CH-8091 Zurich, Switzerland; Division of Pulmonary Medicine, University Hospital Zurich, CH-8091 Zurich, Switzerland; Department of Orthopaedics, Balgrist University Hospital, Forchstrasse 340, CH-8008 Zurich, Switzerland; Department of Pulmonology, University Hospital Zurich, CH-8091 Zurich, Switzerland

**Keywords:** Hip arthroplasty, Lung transplantation, Immunosuppression, Antibiotic treatment, Osteonecrosis, Safety, Complications

## Abstract

**Background:**

In recent years, the number of lung transplants has increased rapidly, with higher quality of life and improved survival rates in transplant recipients, including patients with advanced age. This, in turn, means that more transplant recipients will seek musculoskeletal care to treat degenerative joint disease and also trauma incidents. Safety concerns regarding elective and posttraumatic hip arthroplasty in transplant patients include an increased risk of infection, wound healing problems, periprosthetic fractures and loosening of the implants.

**Methods:**

Clinical outcomes and safety aspects were retrospectively reviewed for five primary total hip arthroplasties (THA) in lung transplant recipients with minimal follow-up of two years at average of 2.6 (2–11) years. Patients were recruited from the Zurich Lung Transplant Center comprising of a cohort of 253 patients between January 1st, 2004 and December 31st, 2013.

**Results:**

All five patients subjectively reported excellent outcomes after THA with a final average Harris Hip Score of 97 (86–100). One 71-year-old patient died 26 months after THA unrelated to arthroplasty. One superficial wound healing disturbance was documented. No periprosthetic fractures, no dislocations, no periprosthetic infections, no further revision surgery, no implant loosening was observed.

**Conclusions:**

In conclusion, THA can be safely and successfully performed even in lung transplant patients under long-term immunosuppressive therapy and polymedication, provided a multidisciplinary approach can be granted.

## Background

In recent years, the number of lung transplants has increased rapidly, with improved survival rates in transplant recipients, including patients with advanced age [1]. Lung transplant patients require triple life-long immunosuppression with steroids, as well as immunosuppressive agents preventing graft rejection. These medications have distinct side effects and can complicate medical and surgical treatment.

Lung transplant patients are vulnerable to medical complications also affecting the musculoskeletal system [1, 2]. Long-lasting and high-dose steroid use can lead to symptomatic osteonecrosis of the bones, particularly the femoral head [3–5]. Severe osteonecrosis can lead to subchondral fractures and collapse of the femoral head following total hip arthroplasty (THA) [5]. Despite the risks of complications associated with lung transplants and other solid organ transplants, the number of transplants continues to rise, and with it, regardless of immunosuppression, an improved post-transplant quality of life and better chances of long-term survival. This, in turn, means that more transplant recipients will seek musculoskeletal care to treat degenerative joint disease and also (osteoporotic) trauma incidents.

Safety concerns regarding elective and posttraumatic arthroplasty in transplant patients include the potential risk of perioperative complications, especially an increased risk of infection and wound healing problems due to life-long immunosuppression [5–7]. There is also an increased risk of periprosthetic fractures and loosening, due to osteoporosis and secondary bone loss [2, 8–10].

With regard to transplant patients, it is still under debate which special medical and surgical management approaches could benefit from closer inspection.

Outcomes of THA have been reported in cardiac [6, 11, 12], liver [6, 7, 13–16], and mostly in renal transplants [5–7, 13, 17–25]. In the literature, there are few guidelines regarding particular perioperative precautions and postoperative care after hip arthroplasty in lung transplant patients [26, 27].

The purpose of this review was to examine the perioperative workup and outcome of THA in lung transplant recipients in the Zurich cohort.

## Methods

This is a retrospective study of all patients who underwent primary THA following lung transplantation at the University Hospital of Zurich between January 1st, 2004 and December 31st, 2013. During this period, in total 253 lung transplantations were done. Five THA in five lung transplant patients were identified (Table 1). Transplantation was performed in the study patients for the following underlying diseases: cystic fibrosis (CF), chronic obstructive pulmonary disease (COPD), idiopathic pulmonary arterial hypertension (IPAH) and lymphangioleiomyomatosis (LAM).

**Table 1:** Patient characteristics

Patient	Sex	Age y	Joint indication	Approach	Immunosuppression	Antibiotic prophylaxis	Hospital stay d	Interval y	F/U y	Subjective hip value %	Harris Hip Score (HHS) points	Perioperative complications	Prosthetic complications
MJ	f	47	Posttraumatic AVN	Hardinge	CYA, MMF, PRED	CIP	7	11.0	11.1	90	86	None	None
SB	f	42	AVN	Hardinge	CYA, MMF, PRED	TZP, TEC, CIP	39	12.2	3.1	100	100	Wound healing disturbance	None
SS	m	23	Posttraumatic AVN	Hardinge	CYA, MMF, PRED	TEC, MER (INN)	16	9.4	2.0	100	97	None	None
MH	f	62	Pathologic Fx	AMIS	CYA, MMF, PRED	TZP, TEC, CIP	11	3.1	2.6	90	100	None	None
FS	f	68	AVN	AMIS	CYA, MMF, PRED	TZP, TEC	8	2.4	2.0	100	95	None	None

*AVN* Avascular necrosis, *Fx* Fracture, *Hardinge* Hardinge approach, *AMIS* Anterior minimal invasive surgery – modified Hueter approach, *CYA* Cyclosporine A, *TAC* Tacrolimus, *MMF* Mycophenolate Mofetil, *PRED* Prednisone, *CIP* Ciprofloxacin, *MER* Meropenem, *TEC* Teicoplanin, *TZP* Piperacillin/Tazobactam, *INN* Amoxicillin/Clavulanic acid, *Interval* Time from lung transplantation to total hip arthroplasty, *F/U* Follow-up time

All patients were prepared well in advance for the THA by their lung transplant specialists. Patients underwent extensive cardiopulmonary testing, in-depth anaesthesia assessment and specific infectious disease workup. They were evaluated for their specific risks due to previous infectious issues including chronic bacterial and viral infections. In general, preoperative IV antibiotic treatment was initiated at least 24 h prior to surgery. Mostly, a dual regimen of piperacillin/tazobactam and teicoplanin was used empirically.

Patients continued on their transplantat-specific, mostly triple immunosuppressant regimen of the following drugs: cyclosporine, mycophenolic acid and corticosteroid pre- and postoperatively. In addition, patients were kept on their usual prophylactic antibiotic (pneumocystis pneumonia (PCP)), antiviral and antifungal agents.

Extremity preparation within the operative suite under laminar flow included electric clipping of hair, three-fold antiseptic scrub with alcohol disinfectant, followed by draping with sterile adhesive polyurethane film (Opsite). Two experienced orthopedic surgeons performed the procedures.

Following surgery, the patients were observed in IMCU or ICU. Postoperative antibiotic prophylaxis was given at least until wound healing confirmed. All patients received standard deep vein thrombosis prophylaxis, including compression stockings and low-molecular weight heparin, continuing for 6 weeks after operation.

Clinical and radiological follow-ups were obtained after 6 weeks, 3, 6, 12 and 24 months by the orthopaedic surgeons, in cooperation with their lung transplant specialists. All multidisciplinary patient charts related to the arthroplasty were analysed. Specific outcome measures included VAS, validated functional Harris Hip Score (HHS), subjective hip value, gait function and complications. All patients had a minimal follow-up of 24 months.

The Institutional Review Board approved the study (KEK-ZH-Nr. 2011–0320).

## Results

Between 2004 and 2013, five primary THAs were performed in 4 females and 1 male lung transplant recipients. Age at transplant was 36.2 (14.2–66.5) years. Three patients with avascular necrosis (AVN) of the femoral head presented markedly limited hip function with severe pain that failed to improve with conservative treatment. One patient suffered from the cut-out of cancellous screws through the femoral head, due to posttraumatic AVN after osteosynthesis of a femoral head fracture with three cannulated cancellous screws. One patient presented a pathologic femoral neck fracture caused by enchondroma. Hence, there was a time interval of 9.4 (2.4–12.2) years between lung transplant and THA. Arthroplasty was performed at an average age of 47.2 (23.6–69.0) years.

All five patients demonstrated relevant medical issues and preoperative comorbidities. ASA physical status was an average value of 2.8 (2–4). Most commonly, the patients suffered from chronic lung allograft dysfunction (CLAD), hypertension, diabetes mellitus, pancreatic insufficiency, chronic kidney disease, chronic transplant rejection and chronic infections (chronic cytomegalovirus (CMV) infection, chronic carrier Noro-virus (3 patients), throat swap multi drug-resistant Pseudomonas (1 patient)).

**Fig. 1:**
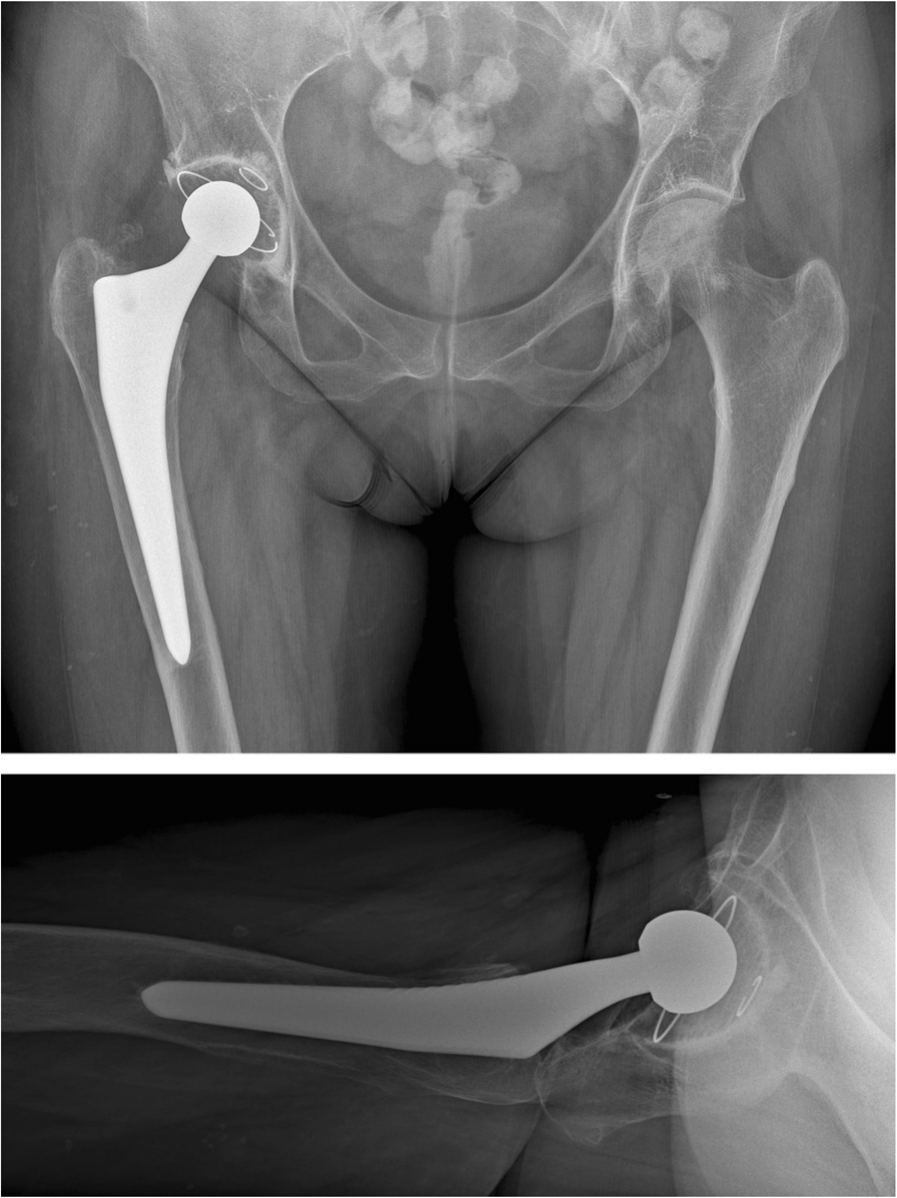
3-year-follow up after THA in 46-year-old lung transplant patient

**Fig. 2:**
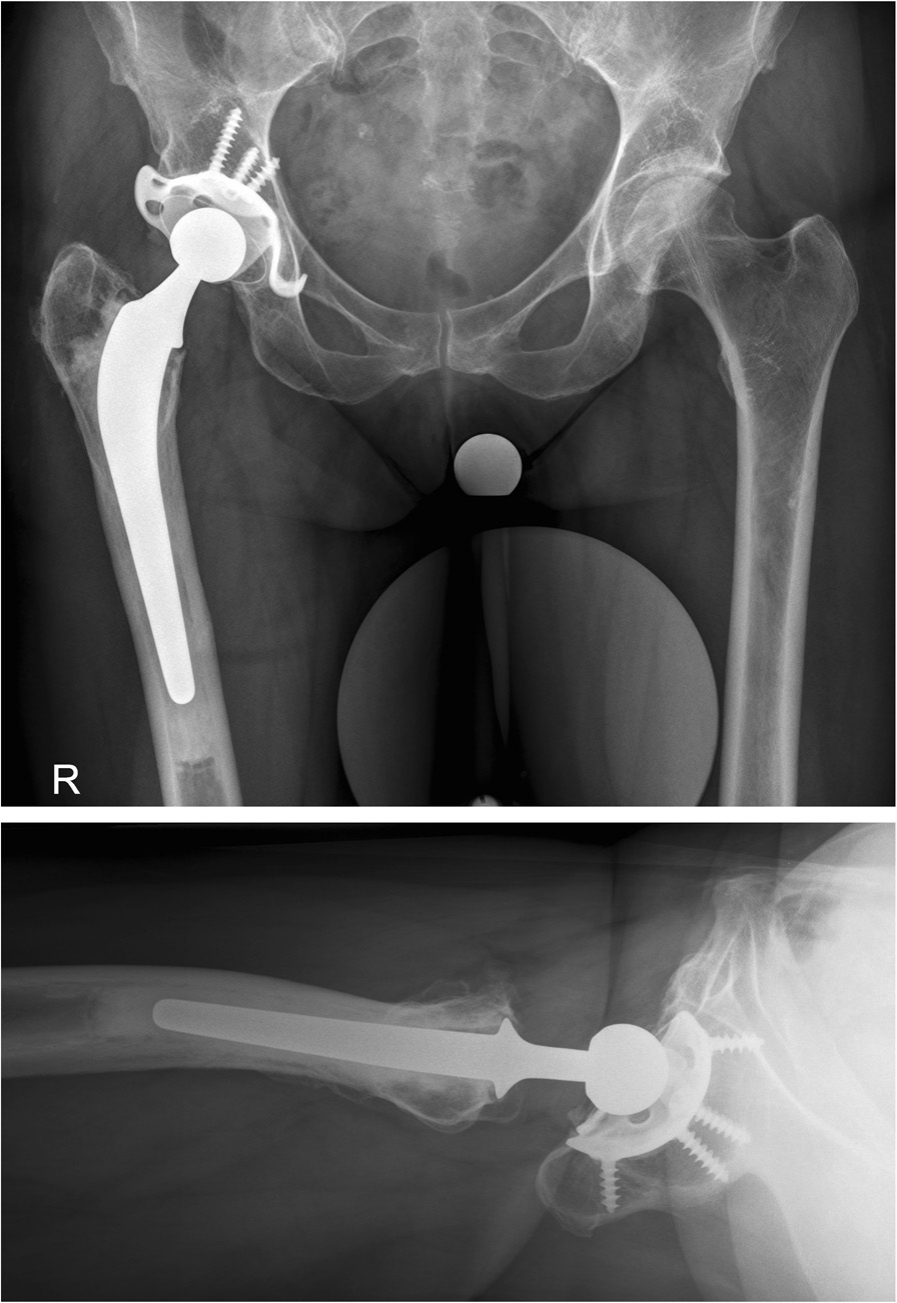
11-year-follow up after THA in 58-year-old lung transplant patient. Ganz reinforcement ring with hook was used because of poor bony quality in the acetabulum

Anaesthesia type was mainly general anaesthesia (four general, one spinal). Two approaches for THA were utilized: anterior “Modified Hueter” approach and lateral Hardinge approach. In more complex reconstructive situations, the lateral approach was favoured, providing a better intraoperative overview and options. In standard situations, an anterior minimal invasive approach was given priority due to its obvious advantages. Acetabular components were cemented using the Apricot highly cross-linked polyethylene cup (Medacta international, Castel San Pietro, Switzerland) and Palacos® R + G bone cement (Heraeus Medical, Wehrheim, Germany)) in all patients (Fig. 1). In one patient, a Ganz reinforcement ring with hook (Zimmer Inc. Warsaw IN, USA) was used in addition (Fig. 2). Femoral stems were uncemented in three cases and cemented in two procedures, due to poor bone quality and pathologic fracture. Femoral heads ranged from size 28 to 32 mm cobalt-chrome heads. Operation time averaged 130 (70–155) min with no intra-operative complications. Average estimated intraoperative blood loss was 400 (200–800) mL. One single redon drainage was used in 80 % of cases. Preoperative haemoglobin was 121 (98–138) gL. Postoperatively, blood transfusions had to be administered in four cases. Full weight bearing was allowed in THA with minimal-invasive anterior “Modified Hueter” approach.

During hospital stay all patients received prolonged prophylactic antibiotic treatment with different medication over 21 (3–35) days. All patients had thromboembolic prophylaxis for six weeks. The postoperative course was complicated in one patient with difficult wound healing with development of a subcutaneous fistula of the absorbable suture after 11 weeks (77 d) requiring operative superficial revision of soft tissue. Length of hospital stay was 11 (7–39) days. No periprosthetic fractures, no dislocations, no periprosthetic infections, no further revision surgery, no implant loosening were observed (Fig. 1). One 71-year-old patient died 26 months after THA unrelated to arthroplasty.

Clinical and radiological follow-up postoperatively was available at an average of 2.6 (2–11) years. All patients were very satisfied and self-reported their subjective hip value of 100 (90–100) %. Average Harris Hip Score was 97 (86–100) points. One patient suffered from mild peritrochanteric pain, without limping.

## Discussion

As the number of patients undergoing lung transplantation increases, there will be a higher frequency of arthroplasties for osteonecrosis, trauma or degenerative osteoarthritis, in order to improve quality of life of transplant recipients.

Perioperative management requires a multidisciplinary approach of trained orthopedic surgeons, lung transplant specialists and specialized anaesthesiologists. It is our policy to directly involve the lung transplant team during the whole hospital stay in the perioperative care of these complex patients.

We observed a low incidence of hip arthroplasty (5 THA in 253 lung transplantations) over a period of 10 years at the Zurich Lung Transplant Cohort that is probably due to the meticulous and close follow-up management of lung transplant patients at our Institution. Furthermore, adaptation of immunosuppression for each individual lung transplant patient is mandatory to titrate to the lowest dose of the immunosuppression to prevent allograft rejection, however, avoid overimmunosuppression causing infection and poor wound healing. Cyclosporine A, mycophenolate mofetil and prednisone were predominantly used. All patients were prescribed daily medication between 5 and 7.5 mg prednisone accordingly 0.1–0.15 mg/kg/day beyond 12 months after transplantation [27]. The risk of osteonecrosis is less than 3 % when the dosage is kept below 15 mg of prednisone per day [12, 28]. Immunosuppressive drugs such as cyclosporine A help to decrease the risk of avascular necrosis [28]. In the literature, prevalence of osteonecrosis of the femoral head after organ transplantation ranges between 2 and 41 % [4, 5, 29]. Potential reasons for this wide variation are differences in steroid regimens and in the sensitivities of screening modalities including inability to capture data (MRI) on asymptomatic patients. However, the authors are unable to estimate the true incidence of femoral head necrosis in the patient cohort of lung transplant recipients. Otherwise, corticosteroids and immunosuppressive medication cause osteoporosis [2]. The loss of bone mineral density (BMD) is most evident in the first year after transplantation, with a fracture rate up to 29 % [2] when high doses of steroids and immunosuppressives are needed to prevent acute organ rejection [8–10]. Most common fracture sides are vertebral compression fractures [2]. In our cohort, we observed one femoral fracture after a cycling accident 6.4 years after lung transplantation, and one pathological fracture after three years because of benign enchondroma in the femoral neck. Our prophylactic medication augmenting bone density includes mainly calcium and vitamin D supplementation, and bisphosphonates based on bone mineral density assessment.

To prevent postoperative infection in patients with triple immunosuppression is of major importance. In our collective, there was one case of superficial wound healing disturbance. After 11 weeks, the patient suffered from small subcutaneous fistula of absorbable suture and was treated with operative spindle-shaped incision followed by an extended course of intravenous antibiotics. The literature reports a higher incidence of periprosthetic infection in transplant patients, with rates varying from no increase, to rates up to 19 % infections in various publications [5, 7, 25]. Vergidis et al. report 12 periprosthetic joint infections in 365 patients (3.4 %) [6]. Most data is mostly available from renal transplant patients. Ledford et al. reported in his collective of 15 THA in 10 lung transplant recipients, no periprosthetic infections or revision surgeries [26].

Wide variance of results and small patient populations make it difficult to establish a definitive consensus of antibiotic treatment in these immunocompromised patients. In the literature, no guidelines regarding type and duration of antibiotic treatment exist. In some studies, antibiotic prophylaxis with cephalosporines was used predominantly for 24 h [14, 26]. In one other study, antibiotic prophylaxis was mainly cephalosporines or vancomycin over 6 weeks [6]. We strongly support prolonged appropriate antibiotic treatment until entire wound healing occurs. Teicoplanin, piperacillin and ciprofloxacin were predominantly used in combination. Drug interaction with immunosuppression and potential side effects should be carefully considered [27]. Duration of postoperative antibiotic treatment averaged 21 (3–35) days. Preoperative intravenous antibiotic treatment was started at least 24 h before surgery.

In our case series, the transfusion rate was very high. Despite the intraoperative blood loss being an average of 400 mL, during the postoperative course four patients received a blood transfusion for symptomatic anemia between the first and 5th postoperative day. The decision to transfuse was based on clinical symptoms and in conjunction with the recommendation by the lung transplant team. First hemoglobin after 24 h counted on average 98 (72–113) gL. High frequency of blood transfusions likely reflects the relative inability of lung transplant patients to tolerate a combination of fluid imbalance and minor fluctuations in hemoglobin levels postoperatively, because of restricted pulmonary function due to impaired lymphatic drainage. Furthermore, preoperative chronic anemia (hemoglobin 123 gL), low body weight (51 kg) and low BMI (19.7 kg/m^2^) in our population in combination with the expected intraoperative blood loss, may result in increased postoperative need for blood transfusion. Patients received, on average 2 units of blood. Other studies report a transfusion rate of 66 % in lung transplant patients [26] and lower rates (39 %) in liver transplant patients [14] after THA. We assume that a low threshold for transfusion is important in this special population, given the inability of lung transplant recipients to tolerate even minor fluctuations in hemoglobin levels postoperatively.

As expected, length of hospital stay in this population was higher than uncomplicated primary THA due to significant comorbidities of the patients. Prolonged intravenous antibiotic treatment and special inpatient recommendations of the lung transplant team to facilitate a safe hospital inpatient stay lead to a longer hospital stay. In our population, we had just a minor complication, nor periprosthetic infections and major medical complications. Responsible medical staff noticed special attention being given to special perioperative precautions in lung transplant patients (Table 2).

**Table 2:** Perioperative precautions relating to arthroplasty in lung transplant patients (adapted from Schuurmans et al. [27])

1	Exhaustion of conservative treatment, interdisciplinary approval of operative treatment, “No standard indications for non-standard patients!”
2	Meticulous preparation of procedure with all involved specialists that may be relevant (including lung transplant specialist, experienced surgeon, specialized anesthetist, experienced intensive care physician for possible postoperative care)
3	Additional intravenous anti-infective treatment for at least one day before and prolonged after arthroplasty
4	Early and consequent laxative treatment to prevent intestinal complications. Avoid opioids.
5	Cautious blood pressure control and accurate fluid management. Arterial hypertension is highly prevalent among lung transplant patients. Preoperatively no restricted fluid intake to avoid hemodynamic instability and renal dysfunction. Fluid overload should be avoided intra-operatively due to impaired lymphatic drainage
6	Low threshold for transfusion postoperatively
7	Strict anti-reflux measures to prevent gastro-esophageal reflux and aspiration
8	Preventive strategies including intensive care unit bed ‘on standby’ after surgery
9	Anticipation of high likelihood of possible complications (kidney failure, hematoma, wound healing disturbance, delirium

In our cohort, one 71-year-old patient died 26 months after THA (4.6 years after lung transplantation) because of progressive CLAD. The only publication from Ledford et al. of a lung transplant patient cohort with THA reported a high mortality rate in 3 patients (33 %) at time of follow-up after 27.5 months [26]. It is difficult to draw specific conclusions based on these data due to the shortened life expectancy could be related to other medical comorbidities and small patient cohort.

To the authors’ knowledge, there are no reports in the literature of THA in lung transplant patients using minimal-invasive anterior approach. In two of our cases, we used this anterior “Modified Hueter” approach without any difficulties intra-operatively. Special care requires precise exposure of the femoral entrance after capsular release avoiding periprosthetic shaft and greater trochanteric fractures due to poor bone quality.

Another controversial technical issue is THA fixation in lung transplant recipients [20]. Generally, patients require triple life-long immunosuppression including steroids. Prolonged corticosteroid use is a significant risk factor for periprosthetic fractures and loosening of the implants due to osteoporosis and secondary bone loss [2, 8–10, 28]. Cemented acetabular and femoral components were used based on intraoperative assessment of weakened bone that will not allow press-fit technique. Since this was the case in all our patients, we cemented all acetabular components; in fact, in one additional patient, an acetabular roof reinforcement ring with hook was used. Femoral stems were uncemented in 3 cases and cemented in 2 procedures. We believe in the benefit of cemented implants, especially in the acetabulum, because of the perpetual steroid medication. Intraoperative acetabular and femoral fractures have to be avoided. Regarding cemented THA, Goffin et al. report on revision-free survival for all implants on 98.8 % at 10 years in renal transplant patients [20]. In contrast, recent evidence suggests that uncemented hip arthroplasty in renal transplant recipients have similar survivorship [5, 17, 30]. Ledford et al. used, in his collective of 15 THA in 10 lung transplant recipients, cementless implants [26]. In our population, no THA dislocation or loosening of the implants were observed during follow-up.

A limitation of our study includes small numbers of patients, midterm follow-up and its retrospective design.

## Conclusions

In conclusion, THA can be safely and successfully performed even in lung transplant patients, provided a multidisciplinary approach can be granted.

## Abbreviations

AMIS, Anterior minimal invasive surgery; AVN, Avascular necrosis; BMD, Bone mineral density; BMI, Body mass index; CF, Cystic fibrosis; CIP, Ciprofloxacin; CLAD, Chronic lung allograft dysfunction; CMV, Cytomegalovirus; COPD, Chronic obstructive pulmonary disease; CYA, Cyclosporine A; F/U, Follow-up; FX, Fracture; HHS, Harris hip score; ICU, Intensive care unit; IMCU, Intermediate care unit; INN, Amoxicillin/Clavulanic acid; IPAH, Idiopathic pulmonary arterial hypertension; IV, Intravenous; LAM, Lymphangioleiomyomatosis; MER, Meropenem; MMF, Mycophenolate mofetil; MRI, Magnetic resonance imaging; PCP, Pneumocystis pneumonia; PRED, Prednisone; TAC, Tacrolimus; TEC, Teicoplanin; THA, Total hip arthroplasty; TZP, Piperacillin/Tazobactam; VAS, Visual analogue scale
